# Nursing interventions to empower the family caregiver of person with lower limb amputation: scoping review

**DOI:** 10.1590/0034-7167-2023-0264

**Published:** 2024-05-03

**Authors:** Diana Fonseca Rodrigues, Fabiana Sofia Ferreira Machado Castanheira, António Luís Rodrigues Faria de Carvalho, Cristina Maria Correia Barroso Pinto

**Affiliations:** I University of Porto, Institute of Biomedical Sciences Abel Salazar. Porto, Portugal; II Center for Health Technology and Services Research and Health Research Network. Porto, Portugal; III Nursing School of Porto. Porto, Portugal

**Keywords:** Amputation, Empowerment, Family Caregiver, Lower Limb, Nursing, Amputação, Empoderamento, Familiar Cuidador, Membro Inferior, Enfermagem, Amputación, Empoderamiento, Familiar Cuidador, Miembro Inferior, Enfermería

## Abstract

**Objectives:**

to map nursing interventions that empower the Family caregiver of the person with lower limb amputation for is role.

**Methods:**

scoping review guided by Joanna Briggs Institute methodology conducted in different databases (including gray literature).

**Results:**

six studies published between 2009 and 2021 were included. Interventions of counselling and support for patients and family; peer support interventions performed by a certified pair; involvement of caregivers or family members in support groups; and key interventions for patient and family caregiver psychological balance. Two studies discussed the importance of caregiver and amputee training and development of coping skills. Another study recommended Interventions of informative support for caregivers regarding care for the amputee and adaptation to home.

**Conclusions:**

results of this review allow the identification of recommendations (guidelines) for practice and recommendations/suggestions for interventions according with identified needs of family caregivers of patients with lower limb amputation.

## RESUMO

**Objetivos:**

mapear intervenções de enfermagem que capacitem o cuidador familiar da pessoa com amputação de membro inferior para seu papel.

**Métodos:**

revisão de escopo orientada pela metodologia do *Joanna Briggs Institute* realizada em diferentes bases de dados (incluindo literatura cinzenta).

**Resultados:**

foram incluídos seis estudos publicados entre 2009 e 2021. Intervenções de aconselhamento e apoio para pacientes e familiares; intervenções de apoio de pares realizadas por uma dupla certificada; envolvimento de cuidadores ou membros da família em grupos de apoio; e intervenções-chave para o equilíbrio psicológico do paciente e do cuidador familiar. Dois estudos discutiram a importância do treinamento do cuidador e do amputado e do desenvolvimento de habilidades de enfrentamento. Outro estudo recomendou intervenções de suporte informativo para os cuidadores com relação aos cuidados com o amputado e à adaptação ao lar.

**Conclusões:**

os resultados dessa revisão permitem a identificação de recomendações (diretrizes) para a prática e recomendações/sugestões para intervenções de acordo com as necessidades identificadas dos cuidadores familiares de pacientes com amputação de membros inferiores.

## RESUMEN

**Objetivos:**

mapear intervenciones de enfermería que capaciten al cuidador familiar de la persona con amputación de miembro inferior para su rol.

**Métodos:**

es una revisión de alcance guiada por la metodología del Instituto Joanna Briggs, llevada a cabo en diferentes bases de datos (incluyendo literatura gris).

**Resultados:**

se tuvieron en cuenta seis estudios publicados entre 2009 y 2021, a saber: intervenciones de asesoramiento y apoyo para pacientes y familiares; intervenciones de apoyo entre iguales realizadas por un par de profesionales certificados; participación de cuidadores o familiares en grupos de apoyo e intervenciones clave para el equilibrio psicológico del paciente y del cuidador familiar. Dos estudios analizaron la importancia de la formación del cuidador y del amputado y del desarrollo de habilidades de afrontamiento. Otro estudio recomendó intervenciones de apoyo informativo para los cuidadores en relación con el cuidado del amputado y su adaptación a la vida hogareña.

**Conclusiones:**

los resultados de esta revisión permiten identificar recomendaciones/directrices para la práctica y recomendaciones/sugerencias de intervenciones según las necesidades identificadas de los cuidadores familiares de pacientes con amputación de miembros inferiores.

## INTRODUCTION

Lower limb amputation is immeasurably disabling for both individual and family. It causes a reduced quality of life (reduced mobility, absenteeism or unemployment, and social isolation), with a profound impact on a person’s mental and emotional health. According to the Amputee Coalition, peripheral vascular disease and diabetes are responsible for more than 54% of lower limb amputations, with 45% of amputations caused by traumatic events and 2% related to cancer^(1)^.

Peripheral arterial disease (PAD), one of the main causes of lower limb amputation, is characterized by a decrease in blood perfusion, which threatens limb viability. Critical ischemia presents with persistent rest pain, and when revascularization of the limb is not achieved, it ends in amputation^(2)^.

Koivunen et al. reported that a person who underwent lower-limb amputation experienced functional, psychological, and social changes, with a significant impact on quality of life^(3)^. Amputation leads to functional limitations that include dependence on daily living activities, changes in balance, decreased mobility, and an increased likelihood of permanent disability^(4)^. The loss of physical, psychological, or intellectual autonomy caused by disease and worsened by the amputation makes it impossible for the person to perform daily life activities independently, thus compromising self-care and making them dependent on someone to perform their daily life activities^(5)^.

In a situation of disability, regardless of its cause, there is a loss of autonomy in performing activities of daily living, leading the person to become dependent and requiring support and help from a caregiver. The experience of caring for a dependent person has become increasingly common in the daily lives of families. This situation brings about contradictory and antagonistic feelings with associated tensions and conflicts. The impact of the reality of the new role of a caregiver occurs when the dependent person and his/her caregiver returns home^(6)^.

Prior to amputation in some situations, the amputee already presents some type of dependence that is aggravated by the amputation. The greatest difficulties for caregivers include mobilising and transferring the person, the appropriate use of body mechanics, communication management, and therapy management, among others^(7)^. Early intervention is fundamental, as well as the promotion of family caregiver empowerment^(7-8)^. This evolution has provided benefits and health gains^(8-9)^.

To assume the role of family caregivers, it is essential to have knowledge about the disease of the person cared for and to acquire new care skills^(10)^. The transition to the role of caregiver is a critical period in learning skills. The caregiver needs to acquire knowledge and skills on how to perform all activities of daily living of the dependent person regarding mobilisation, transfer, medical assistance at home, and care at home^(9,11)^. The process of transitioning to the role of the caregiver is complex and includes different needs throughout its development. The nurse’s role is essential and should be oriented towards meeting the needs of the family caregiver and the person being cared for^(7)^.

Providing care for a dependent family member is an exhausting process, with an associated burden in various areas, with various associated problems and challenges, leading to negative consequences for the family caregiver. Nurses, as health professionals, have a privileged and close role, with specific skills that allow them to develop and implement structured and contextualized interventions, allowing the people involved in the process to have more positive and favorable experiences^(12)^.

The needs of caregivers vary depending on the transition phase that is found in both the caregiver and the dependent person, so the assessment of needs should consider all areas of care, family relationships, physical and emotional self-care, and knowledge and skills should also be evaluated^(13)^. The acknowledgement of the needs and competencies of the informal caregiver of a dependent person, to capacitate them in taking care of their relatives before home discharge, can help decrease their burden and decrease the number of hospital readmissions^(14)^. To ensure continuity of care and higher quality care for the dependent person and to promote the satisfaction of the caregiver’s and the patient’s needs, the integration of the caregiver in intervention programs is fundamental^(7-8,15)^.

The development of intervention programs for caregivers aims to minimize or prevent the possible negative consequences that may arise from caregiving. Some authors advocate the creation of educational manuals for caregivers directed to specific situations such as stroke^(11)^. This type of strategy, as well as the use of simulation, is intended to improve the quality of life, well-being, and burden of caregivers while promoting a more optimised and goal-oriented care delivery^(10-11,16)^.

In this context, there is little knowledge about the interventions used by nurses to empower the family caregiver of a person with lower-limb amputation. Nurses must implement interventions to respond to the needs of family caregivers of a person with lower limb amputation, helping reduce the burden of care. Therefore, there is a need to map evidence to establish the current extent, scope, and nature of this emerging field of research. This scoping review will contribute to the dissemination of research results, the identification of possible gaps in knowledge in this area, and the need for future research by providing a detailed description and summary of the available information^(17)^.

A preliminary search was conducted on 10 January 2022 to identify scoping reviews and systematic reviews in the MEDLINE Complete (EBSCO), CINAHL Complete (EBSCO), and Joanna Briggs Institute (JBI) Database of Systematic Reviews and Implementation Reports databases. No literature review (published or forthcoming) was found on the topic of this scoping review. Given this fact, the aim of this scoping review was to identify and systematize the existing knowledge in both the published literature and gray literature on educational or formative nursing interventions aimed at empowering the family caregiver of a person with lower limb amputation for his/her role.

The following research question was formulated to guide this study: “What nursing interventions, educational or formative, empower the family caregiver of the person with lower limb amputation for their role?”.

## OBJECTIVES

To map nursing interventions that empower the Family caregiver of the person with lower limb amputation for is role.

## METHODS

The scoping review methodology is used in each area of research to map available concepts, sources, and evidence or to identify possible gaps related to the topic under study.

The development and preparation of this scoping review followed the recommendations of the Joanna Briggs Institute^(17-18)^. The study was guided by the Preferred Reporting Items for Systematic Reviews and Meta-analyses Extension for Scoping Reviews (Prisma-ScR) checklist to ensure transparency and rigor^(19)^. A research protocol was prepared and registered in the Open Science Framework (http://osf.io/su8kb)^(20)^.

### Inclusion criteria

The inclusion criteria were defined based on the research question and the type of evidence added according to the PCC (Participants, Concept and Context) strategy.

Participants - Family caregivers of a person with lower limb amputation, aged over 18 years.

Concept - Studies that report nursing interventions within the scope of therapeutic education or interventions involving caregiver empowerment. Studies on nursing interventions in therapeutic education aimed at wound care or stump preparation for prosthesis placement were also excluded.

Context - Studies involving caregivers of hospital inpatients returning home. Studies involving primary health care or studies directed at family caregivers of patients with lower limb amputation admitted to long-term care facilities were excluded.

Type of evidence - Quantitative, qualitative, and mixed studies, primary studies, systematic reviews, books, theses, dissertations, and guidelines, published in indexed sources or in gray literature were included. Other texts such as opinion papers and reports were also considered.

The included literature will be limited to studies written in Portuguese, English, and Spanish, since these are the languages in which reviewers are proficient.

### Search strategy

The search strategy used was the three-step strategy proposed by the Joanna Briggs Institute^(15)^ to find published and unpublished studies that answered the review question. In the first stage an initial search limited to the CINAHL Complete (EBSCO) and MEDLINE Complete (EBSCO) databases was conducted using the Boolean phrase (Caregiver*) AND (“Lower Limb*”) AND (“Amputat*”) AND (nursing OR intervention*). Followed by an analysis of the index terms and text words used in the titles and abstracts of the obtained articles used in the selected databases.

In the second stage, the index terms and keywords identified in the initial review results were combined using a full search strategy adapted for all included databases. The search included both published and unpublished studies in peer-reviewed databases and gray literature sources. The search strategy was refined with the assistance of a librarian.

The search strategy was then adapted for each included information source, and a complete search of all databases was undertaken on April 4, 2022, presented in Chart 1.

**Chart 1:** Search strategies according to databases

Search Strategy	Databases	Results
TI ( (Caregiver^*^ OR “Care Giver^*^” OR “Carer^*^”) AND (“Lower Extremit^*^” OR “Lower Limb^*^” OR “Membrum inferius”) AND (“Amputat^*^”) AND (nursing OR intervention^*^ OR strateg^*^ OR program^*^) ) OR SU ( (Caregiver^*^ OR “Care Giver^*^” OR “Carer^*^”) AND (“Lower Extremit^*^” OR “Lower Limb^*^” OR “Membrum inferius”) AND (“Amputat^*^”) AND (nursing OR intervention^*^ OR strateg^*^ OR program^*^) ) OR AB ( (Caregiver^*^ OR “Care Giver^*^” OR “Carer^*^”) AND (“Lower Extremit^*^” OR “Lower Limb^*^” OR “Membrum inferius”) AND (“Amputat^*^”) AND (nursing OR intervention^*^ OR strateg^*^ OR program^*^) )	MEDLINE Complete (EBSCO)	10
	CINAHL Complete (EBSCO)	7
	Supplemental Index (EBSCO)	7
	OpenAIRE (EBSCO)	7
	Complementary Index (EBSCO)	3
	Academic Search Complete (EBSCO)	3
	SPORTDiscus with Full Text (EBSCO)	2
	Gale Health and Wellness (EBSCO)	1
	OAIster (EBSCO)	1
	Science Direct (EBSCO)	2
	Scielo (EBSCO)	1
	RCAAP (via EBSCO)	5
TITLE-ABS-KEY ((caregiver^*^ OR “Care Giver^*^” OR “Carer^*^”) AND (“Lower Extremit^*^” OR “Lower Limb^*^” OR “Membrum inferius”) AND (“Amputat^*^”) AND (nursing OR intervention^*^ OR strateg^*^ OR program^*^))	Scopus	20
TS=((Caregiver^*^ OR “Care Giver^*^” OR “Carer^*^”) AND (“Lower Extremit^*^” OR “Lower Limb^*^” OR “Membrum inferius”) AND (“Amputat^*^”) AND (nursing OR intervention^*^ OR strateg^*^ OR program^*^))	Web of Science	20
(Caregiver^*^ OR “Care Giver^*^” OR “Carer^*^”) AND (“Lower Extremit^*^” OR “Lower Limb^*^” OR “Membrum inferius”) AND (“Amputat^*^”) AND (nursing OR intervention^*^ OR strateg^*^ OR program^*^)	Google Schoolar	17
	Trip	5

In the third stage, the bibliographic references of the included studies were consulted and analysed to include potential studies in the review. Two studies were included from the bibliographic references.

### Study selection

From a search conducted by two independent researchers, 111 references were found and exported to Mendeley V1.19.8 Software (Mendeley Ltd., Elsevier), where duplicates in the literature were excluded. Prior to the start of selection, the research team met the established inclusion and exclusion criteria. The literature selection was conducted in two stages. In the first stage, titles and abstracts were read and studies that did not meet the predetermined inclusion and exclusion criteria were eliminated. In the second step, the full text of the literature was analyzed to verify its adequacy to the previously defined inclusion criteria. In each step, two independent researchers performed selection. Literature evaluation and selection were performed according to the Preferred Reporting Items for Systematic Reviews and Meta-Analyses (PRISMA) flow diagram^(21)^. Since this was a scoping review, the methodological quality of the selected studies was not analyzed^(17)^.

### Data Extration

Data extraction was performed by two independent researchers using an instrument developed according to the recommendations of the Joanna Briggs Institute. The selected literature was analyzed according to the following specific details: author, year of publication, country of origin, population, methodology, type of nursing intervention, duration and frequency of intervention, and site of intervention. It was not necessary to contact authors for missing information.

The study classification according to the level of evidence was based on the Oxford Center Evidence Based Medicine^(22)^, and the studies were classified according to the research design into ten levels:1a, 1b, 2a, 2b, 3a, 3b, 4, and 5, with the highest level of evidence 1a and 5 the lowest.

## RESULTS

**Figure 1:**
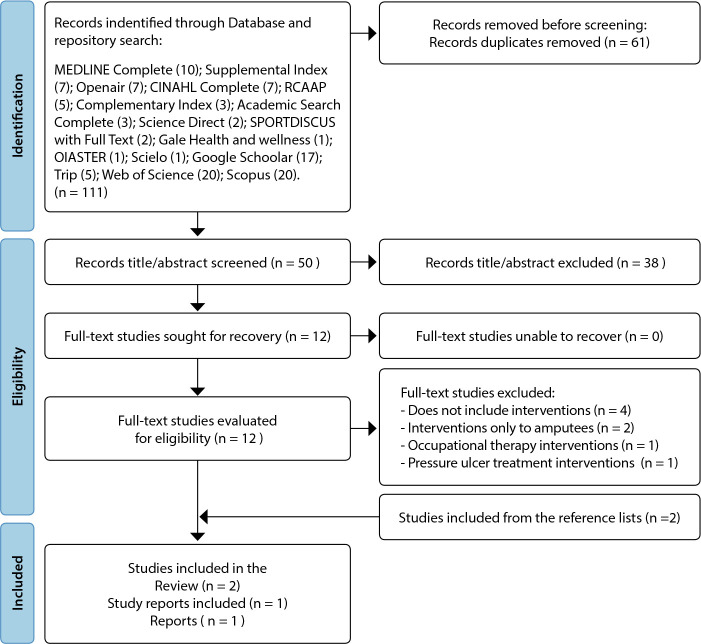
Flow diagram according to PRISMAScR guidelines (adapted)

From the search made in the various databases and repositories meeting the established inclusion and exclusion criteria, 111 bibliographic references were identified as being potentially relevant. the literature selection is shown in the flowchart in Figure 1.

A total of 111 potentially relevant studies were identified for study selection, after the duplicates removed (n=61), the remaining 50 studies were evaluated by title and abstract. A total of 12 studies remained for full-text reading, and only four studies fulfilled the inclusion criteria. Another 2 studies were included from the reference lists of the four included studies, and a total of six studies were included in this review.

### Studies Characteristics

The six studies included in this review came from five countries, two studies conducted in Portugal, and one each in Australia, USA, Brazil, and Iran. The studies were published between 2009 and 2021; five studies were published in English, and one study in Portuguese. The study had a qualitative design (n=2), quantitative design (n=1), quantitative non-experimental design (n=1), evidence review guidelines (n=1), and guideline reports based on a systematic review (n=1). The participants in the studies ranged from 87 to 232 family caregivers of lower limb amputees, and in two of the studies, the participants were not referred. The characteristics of the included studies are shown in Chart 2.

**Chart 2:** Grid for data extraction

Author(s) (year) Country	Methodology (Study Design)	LE^*^	Participants	Interventions	Duration/ frequency of intervention	Intervention Context
Tivey et al. (2015)^(23)^ Australia	Review of Standards evidence or Guidelines.	5	Patients and family members	Set of recommendations: - Adequate and comprehensive psychological and emotional support services for patients and family members. - Peer support and Support group	Start in the preoperative and prolong in the postoperative period	Hospital and home
Randolph et al. (2017)^(24)^ USA	Clinical practice guidelines based on a systematic review of clinical and epidemiological evidence.	5	Amputees and family members	Set of recommendations: - Facilitate the visit of peers soon after surgery to build a support and support network. - Include family members in the patient’s rehabilitation. - Build and maintain trust, respect and support with the patient and his family.	As early as possible and with follow-up visits,	Hospital and home
Costa et al. (2021)^(25)^ Portugal	Longitudinal Study	4	110 family caregivers of amputees due to diabetic foot with type 2 diabetes	- Intervention of Help Relationship in the nursing domain, contributing to the satisfaction of the caregiver’s needs. - Nursing intervention (within the scope of inform - offering systematized information to the caregiver).	According with nurses’ decision (optimal time to begin)	Hospital and home
Costa et al. (2018)^(26)^ Portugal	Longitudinal study	4	110 family caregivers of type 2 diabetic patients with diabetic foot undergoing amputation surgery.	- Interventions aimed at training patients and caregivers to collaborate in care. - Social support interventions for the patient and caregiver. - Instrumental support interventions for the patient and caregiver.		Return home
Foss et al. (2009)^(27)^ Brazil	Prospective Study	4	87 family caregivers of lower limb amputees	- Interventions of informative support for the family caregivers about: care for the amputee, adaptations to the home, possibilities of leisure and adequacy of the means of locomotion of the amputee.		Hospital and home
Ganjparvar et al. (2016)^(28)^ Iran	Cross-sectional study	4	232 family caregivers (wives) of bilateral lower limb amputees	- Interventions aimed to family caregivers training. - Training amputees and family caregivers regarding the social, emotional, and psychological aspects of amputees’ life. - Teach family caregivers coping strategies for psychosocial problems. - Social support for amputee and caregiver. - Promote rehabilitation Programs.	Long life Support	Hospital and home

LE^*^ - Level of evidence.

### Summary of results

As shown in Chart 2, the six studies included in this review contained information about interventions that can be developed by nurses as health professionals to help family caregivers of persons with lower limb amputations in the transition to their role.

Two studies^(23-24)^ included evidence-based information and recommendations for clinical practice regarding amputee care and work as guidelines for health professionals including nurses, physicians, and other professionals working with amputated individuals and families. This set of recommendations focuses on support services for patients and families, peer network support, and the integration of families in the rehabilitation program with the amputee. This type of intervention should begin as soon as possible and be prolonged by the pos operatory with follow-up visits.

Foss et al.^(27)^ recommended interventions of informative support for family caregivers: care for the amputee, adaptations to the home, possibilities of leisure, and adequacy of the amputee’s means of locomotion according with the amputee caregivers identified needs. Nurse interventions of informative support offering systematized information to family caregivers have been suggested in another study^(25)^.

Interventions that address the training of the family caregiver and the person with lower limb amputation are considered important by evidence^(26,28)^. Training family caregivers and amputees to collaborate in care is considered important in one of the included studies^(26)^. Another study alluded to the importance of training family caregivers and amputees regarding the social, emotional, and psychological aspects of amputees’ life^(28)^.

Promoting a rehabilitation program that includes a person with lower limb amputation and their family caregiver is a recommended intervention presented in two studies included in the review^(24,28)^.

The development of interventions for social support to help a person with lower limb amputation and the caregiver make a healthy transition is mentioned in the evidence included in this review^(26,28)^. Interventions of instrumental support to help the person with lower limb amputation and the family caregiver in the return home should by used by nurses^(26)^.

Nurses as health providers should teach family caregivers coping strategies for psychosocial problems and to cope with the situation of caring for a person with lower limb amputation according with Ganjparvar^(28)^.

## DISCUSSION

This scoping review aimed to map and analyze the interventions led and/or implemented by nurses to empower the family caregiver of a person with lower limb amputation. We found studies conducted in different countries. Two of the studies found in the review were guidelines for practice^(23-24)^. In both studies, it is evident that interventions aimed at guiding and preparing (empowering) family caregivers are fundamental because they provide them with the appropriate conditions for them to take care of their dependents. However, for this to happen, it is necessary to have teamwork, an interprofessional work that seeks to identify and intervene according to the needs of each patient and caregiver^(29)^.

According to the evidence of the investigated studies, interventions for informative support are very important for family caregivers^(25,27)^. Informative support about the care of the amputee, adaptation to the home, possibilities of leisure, and adequacy of the means of locomotion of the amputee can help improve the quality of life of family caregivers and amputees^(27)^. Costa et al.^(25)^ referred to the need for nurse interventions of informative support for family caregivers of amputees according to the identified needs. Many caregivers experience a high level of burden. Nurses should invest more in informal caregiver information/training to prevent burden and improve the quality of care. It is important to highlight that in addition to information, caregivers must be prepared throughout hospitalization to care for the dependent person^(30)^.

Peer Support and Peer support group interventions for amputees are considered important to help amputees and family caregivers cope with this new reality. This intervention should start in the preoperative period and prolong the postoperative period with the visit of peers soon after surgery with follow-up visits to help construction of support and support networks for the amputee and caregiver^(23-24)^. The early involvement of family members and contact with other amputation patients are important for the psychological balance of the amputee^(24)^. Peer-to-peer help groups, under the guidance of a professional, are inexpensive methods that provide space for sharing experiences without great human and physical resources. They provide a space for knowledge about the use of coping strategies, encourage the mobilization of aid, create perspectives, and reduce social isolation. In people with dependence, this type of intervention has been shown to result in the acquisition of knowledge by the caregiver, with improvement in psychological well-being, reduction of burden, and a decrease in hospitalization periods^(31)^.

Caring for a person with lower limb amputation has a negative impact on caregivers’ physical, mental, and emotional health. Psychological and emotional support interventions are essential for both patients and families, and should begin in the acute phase/preoperative phase and continue if required as part of lifelong management^(23)^. Personal factors, such as emotional and physical health, can compromise the performance and adaptation of the relative to play the role of a caregiver. Psychological Counselling is something that family members need to support and understand the repercussions of the disease in the family context during the transition process^(32)^. Emotional support works have a protective factor regarding the quality of life of the caregiver, which reduces the negative aspects of care by the formation of groups of caregivers who experience the same reality or are developed through qualified listening^(33)^. Nurses have part of a professional category whose main focus is care, and must consider the individual as a whole, considering that the psychological, biological, and social aspects are all interconnected and interfere with the health of individuals^(33)^.

Evidence shows that it is important for nurses as health professionals to teach family caregivers coping skills; preparing them for potential psychosocial problems or situations may increase quality of life in both patients and caregivers^(28)^. Training amputees and their caregivers regarding the psychological, emotional, and social aspects of amputees’ lives might lead to better adjustment to day-to-day life and lower hospitalization rates^(28)^. Emotion-focused coping is an intervention strategy that may improve depressive symptoms, anxiety, quality of life, and caregiver burden. Developing coping strategies is essential for managing psychological distress in caregivers^(34)^. Caregivers who use emotion-focused adaptive coping and problem-focused strategies can adapt to the caregiver’s role^(35)^. Nurses must identify the family’s main difficulties, needs, and potential in performing their new role and identify which strategies are used as well as variables that may influence their mobilization. Nursing care centered on the family as a unit of care should promote empowerment according to the transitions that occur throughout the life cycle^(36)^.

According to evidence, it is vital to provide social support interventions that allow patients and caregivers to collaborate in the process of caring, and social support can moderate the effect of caregiving burden^(26,28)^. The burden imposed by the caregiver’s role can be reduced if the social support to these caregivers is improved, and interventions from nurses should devise state interventions, programs, and aid directly to the caregivers to help them ease their role in improving health results^(37)^. Social support is substantially correlated with caregivers’, quality of life. When providing care, caregivers should be encouraged to request assistance from friends and family members, especially when caregivers are long-term care or unemployed^(38)^. Satisfaction with social support influences quality of life. The greater the satisfaction with social support, the greater the caregivers’ quality of life^(39)^.

The literature recommends that health services assess the caregivers of amputees and that health policies should provide instrumental support to allow amputees and caregivers to collaborate in the process of caregiving^(26)^. Instrumental support includes helping with daily living needs and housework^(40)^. Caregivers need family instrumental support and having someone to help them in more physically demanding care, especially for older and/or sick caregivers. Instrumental support from paid and nonprofessional caregivers can help caregivers in caring tasks and household chores^(41)^. On days, caregivers received instrumental support and the daily odds of depressive symptoms decreased^(42)^.

Training interventions for family caregivers and amputees to prepare them for care and the aspects of amputees’ lives are considered important in two of the included studies^(26,28)^. During caregiver training, intervention is recommended as early as possible with the amputee, including family members during rehabilitation^(24)^. Promoting rehabilitation programs can help address the needs of amputees’ family members. Interventions to train family caregivers are fundamental to the acquisition of knowledge and strategies that help reduce their burden^(16)^. The needs and difficulties of the amputee’s family caregiver are diverse and vary according to the transition phase in which the family caregiver and amputee person are in. It is crucial to identify and assess these needs and difficulties in understanding the most appropriate interventions. The informal caregiver training process is central to identifying training needs by evaluating the capacity to care for a person with self-care dependence^(13)^.

Evidence seems to show that intervention programs can decrease and prevent burden, with a positive impact on care and improvement in psychological status, reflecting a decrease in rehospitalizations^(8,15)^. The various intervention programs that are directed to family caregivers cover several typologies: educational interventions, psychoeducational or psychotherapeutic interventions, support groups, support or self-help, interventions directed to new technologies, multimodal programs (combining various interventions), formal support (community services), and economic or legal aid^(8,15)^.

The family caregiver is undoubtedly a partner in caring for a person who has lost the ability to perform self-care activities. It is the responsibility of the health services and nurses, in particular, to prepare, accompany, and provide support to the caregiver to meet the needs of the person with dependency. Before intervention, nurses assessed the caregiver and the person being cared for in order to choose an appropriate intervention plan for each context and situation. Scientific evidence has shown that there are several paths and strategies for empowering family caregivers^(43)^.

### Study limitations

The limitation of this study was the inclusion only of articles in three languages Portuguese, English and Spanish (proficiency domain of the researchers), so some relevant studies may have been omitted.

### Contributions to Nursing

The present study brings an overview of scientific evidence and existing knowledge about interventions led and/or implemented by nurses to empower the family caregiver of a person with lower limb amputation in the transition for their role. This review can provide valuable insights, leaving a set of recommendations/guidelines for nurses and contributing to the development of nursing interventions.

This scoping review can contribute to the development of new studies that demonstrate the impact and importance of nursing interventions to empower the family caregiver of a person with a lower limb amputation in their role. These interventions can minimize the burden of caregiving by helping caregivers develop strategies to cope with the adversities of caring for a person with lower limb amputation.

## CONCLUSIONS

This scoping review provides a comprehensive overview of nursing interventions that empower the family caregiver of the person with lower-limb amputation. This search provided insights into the limited literature in this area. This study allows us to identify recommendations/guidelines for practice and suggestions for interventions based on the identified needs of the family caregivers of amputees.

Family caregivers are an important part of the care network; therefore, they require support to provide that care. Before home discharge of a person with lower limb amputation, it is important to acknowledge the needs and competences of the caregivers to capacitate them in looking after their relatives, decreasing the burden of care and hospital readmissions. Knowing caregivers’ needs may enable the development of appropriate interventions to address these needs and reduce the negative consequences of family care for people with lower limb amputation.

These findings will contribute to a broader knowledge of nursing interventions to empower family caregivers of persons with lower limb amputation. Future research requires larger-scale studies with longer-term follow-ups and validated measurement instruments to examine the effects of these interventions in family caregivers.
